# Soluble PD-L1 changes in advanced non-small cell lung cancer patients treated with PD-1 inhibitors: an individual patient data meta-analysis

**DOI:** 10.3389/fimmu.2023.1308381

**Published:** 2023-11-23

**Authors:** Takashi Shimizu, Eisuke Inoue, Ryotaro Ohkuma, Shinichi Kobayashi, Takuya Tsunoda, Satoshi Wada

**Affiliations:** ^1^Department of Clinical Diagnostic Oncology, Clinical Research Institute for Clinical Pharmacology and Therapeutics, Showa University, Tokyo, Japan; ^2^Clinical Research Institute for Clinical Pharmacology and Therapeutics, Showa University, Tokyo, Japan; ^3^Showa University Research Administration Center, Showa University, Tokyo, Japan; ^4^Division of Medical Oncology, Department of Medicine, School of Medicine, Showa University, Tokyo, Japan; ^5^Department of Pharmacology, School of Medicine, Showa University, Tokyo, Japan; ^6^Pharmacological Research Center, Showa University, Tokyo, Japan

**Keywords:** soluble PD-L1, advanced non-small cell lung cancer, PD-1 inhibitors, individual patient data meta-analysis, biomarker

## Abstract

**Introduction:**

Currently, first-line immune checkpoint inhibitors (ICIs), including programmed cell death protein-1 (PD-1) inhibitors, are utilized as monotherapy in advanced non-small cell lung cancer (NSCLC) patients with high programmed death ligand-1 (PD-L1) expression (≧50%). Pre-treatment or post-treatment serum soluble PD-L1 (sPD-L1) has been identified as a potential biomarker for assessing ICI efficacy through fixed-point observations. However, existing studies on sPD-L1 changes have produced inconsistent results or have had sample sizes too small to detect clinically meaningful effect sizes. To elucidate the role of sPD-L1, we conducted a collaborative individual patient data meta-analysis of PD-1 inhibitor treatments.

**Methods:**

We conducted a thorough search of articles in PubMed via Medline, Embase, Scopus, and Cochrane databases from inception to October 20, 2023. Trials were deemed eligible if they contained individual datasets for advanced NSCLC patients, including data on overall survival (OS)/progression-free survival (PFS), as well as pre- and post-treatment sPD-L1 levels after 3-4 cycles of PD-1 inhibitor treatments. Our analysis focused on patients who completed 3-4 cycles of PD-1 inhibitor treatments. The primary outcome measure was OS/PFS, and we assessed changes in sPD-L1 concentration pre- and post-treatment through ELISA analyses.

**Results:**

From our search, we identified a potential seven trials, encompassing 256 patients. Among these, two trials with 26 patients met the criteria for inclusion in our primary analyses. Over a median follow-up period of 10 months, pooled univariate analysis revealed that increases in sPD-L1 levels during PD-1 inhibitor treatment were not associated with OS (HR = 1.25; CI: 0.52–3.02)/PFS (HR = 1.42; CI: 0.61–3.30) when compared to cases with sPD-L1 decreases. Subgroup analyses indicated that the impact of sPD-L1 changes on overall mortality/progression-related mortality remained consistent regardless of gender, age, or the type of treatment (nivolumab or pembrolizumab).

**Conclusion:**

Our findings suggest that changes in sPD-L1 levels during PD-1 inhibitor treatment do not significantly influence the prognosis of advanced NSCLC patients, regardless of gender, age, or treatment type. Continuous monitoring of sPD-L1 may not offer significant advantages compared to fixed-point observations.

## 1 Introduction

Non-small cell lung cancer (NSCLC) represents the majority of lung cancer cases, which is the leading cause of cancer-related deaths. Most NSCLC cases are diagnosed with distant metastases (1). Therefore, effective treatment in advanced NSCLC cases is crucial (2). After randomized phase 3 trials, nivolumab and pembrolizumab, which are monoclonal antibodies against programmed cell death protein 1 (PD-1), have become a standard treatment for patients with advanced NSCLC (3–5). The prevalence of programmed death protein ligand-1 (PD-L1) expression in NSCLC tissues ranges from 24% to 60%, and tumor PD-L1 expression appears to be a potential biomarker for predicting the effectiveness of immune checkpoint inhibitors (ICIs). Currently, these anti-PD-1 inhibitors have been used as monotherapy in the first-line treatment of advanced NSCLC patients with high PD-L1 expression (≥50% tumor cells) and no EGFR, ALK, or ROS1 aberrations (6). Obtaining tumor tissues can be challenging in advanced NSCLC patients. Tissue analysis is much less suitable for therapy monitoring compared to serum-based assays. Recently, pre-treatment or post-treatment soluble PD-L1 (sPD-L1) in plasma or serum has been reported as potential biomarkers for monitoring ICI therapy in NSCLC patients (7–13). However, few reports have examined whether changes in sPD-L1 over time can serve as a biomarker of ICI efficacy.

Over the past few years, several studies have examined the association between pre-treatment or post-treatment sPD-L1 levels and prognosis in various cancers. In 2023, Széles et al. conducted a meta-analysis to evaluate the correlation between pre-treatment sPD-L1 and survival in a wide range of human malignancies (14). The pooled overall estimate indicated that sPD-L1 is a significant indicator of shorter OS in various cancers, with the strongest association observed in NSCLC. However, when it comes to sPD-L1 changes, previous studies have yielded inconsistent results, possibly due to small sample sizes or a lack of clinically meaningful relationships.

To determine the role of sPD-L1 changes, we conducted a collaborative individual patient data meta-analysis of PD-1 inhibitor treatments, using individual patient data from two of the seven trials.

## 2 Materials and methods

### 2.1 Study design and outcomes

In this meta-analysis, we identified relevant trials by systematically searching MEDLINE, Scopus, Embase, and Cochrane library from database inception to October 20, 2023. Trials were eligible if they included individual datasets of advanced NSCLC patients, encompassing overall survival (OS)/progression-free survival (PFS) and soluble PD-L1 (sPD-L1) levels before and after 3-4 cycles of PD-1 inhibitor treatments. The search terms used were as follows:

1. Soluble PD-L1
2. Non-small cell lung cancer
3. Immune checkpoint inhibitor
4. PD-1 inhibitor
5. Overall survival/progression-free survival
6. Excluding animals

Two independent authors (TS, SW) extracted data by thoroughly reviewing full-text articles. Each trial’s eligibility was determined by evaluating its protocol, methods, and the availability of individual patient data. To control for sources of variability within and between studies, a standardized definition of patients for inclusion in the analyses was employed, and this was explored further through prespecified subgroup analyses. The primary objectives of this meta-analysis were to estimate the impact of sPD-L1 changes on OS/PFS in advanced NSCLC patients, with the prespecified primary outcome being OS/PFS.

### 2.2 Data analysis

We aggregated individual participant data, including relevant characteristics such as age, sex, type of PD-1 inhibitor treatment (nivolumab or pembrolizumab), pre/post-treatment serum sPD-L1 concentration, follow-up period, and overall mortality during each trial. Patients with missing data on post-treatment serum sPD-L1 concentration were excluded. To accommodate multiple subdivisions of the data, only summary effect estimates are presented, along with 95% confidence intervals (CIs).

For the primary comparisons, significance was defined as a two-sided p-value less than 0.05. Results are reported as mean ± standard deviation (SD). Linear correlation analysis was conducted using Pearson correlation. Statistical tests were performed, and figures were created using GraphPad Prism 8.4.3 software (GraphPad Software Inc., San Diego, CA, USA). It is important to note that this database is a research database and did not involve accessing or processing patient-identifiable information; therefore, ethical approval was not required.

### 2.3 Role of the funding source

The funders of the study had no involvement in the study design, data collection, data analysis, data interpretation, writing of the report, or the decision to submit it for publication.

## 3 Results

### 3.1 Search and selection

We initially identified potentially seven trials comprising 256 patients using the search criteria mentioned above. Subsequently, two trials involving 26 patients met the eligibility criteria for inclusion in our primary analyses. Five trials involving a total of 230 patients were not included in the primary analyses as they were unable to provide relevant data.

### 3.2 Baseline characteristics of included studies

**Table 1** summarizes the baseline characteristics of the two included articles. One article was retrospective, while the other was prospective. All studies utilized ELISA assays by R&D Systems (Wiesbaden, Germany) to determine sPD-L1 concentrations. The mean age of participants in these trials was 71.2 years (SD 9.4), with 8 (31%) being female. The mean sPD-L1 concentrations (pg/ml) before and after 3-4 cycles of PD-1 inhibitor treatments were 139 (SD 223) and 144 (SD 213), respectively, with no significant difference (P=0.94). The mean sPD-L1 fold change was 1.39 (SD 0.67).

**Table 1:** Serum sPD-L1 changes before and after 3-4 cycles of PD-1 inhibitors treatment in advanced non-small cell lung cancer (NSCLC) patients.

Author (year)	Study site/type	Type of treatment	No. of patients (female)	Age (mean ± SD, year)	Follow-up period (mean ± SD, months)	Outcome	Pre-treatment sPD-L1 (mean ± SD, pg/mL)	Post-treatment sPD-L1 (mean ± SD, pg/mL)	sPD-L1 fold changes (mean ± SD)
Ando et al., 2019 (7)	Japan/Retrospective	Nivolumab 4, Pembrolizumab 3	6 (1)	69.7 ± 4.5	15.6 ± .7	OS/PFS	489 ± 26.6	476 ± 228	0.99 ± 0.21
Castello et al., 2020 (8)	Italy/Prospective	Nivolumab 13, Pembrolizumab 7	20 (7)	71.7 ± 10.4	10.3 ± 9.2	OS/PFS	33.5 ± 14.5	43.8 ± 13.4	1.51 ± 0.72

### 3.3 sPD-L1 fold changes do not predict OS/PFS in NSCLC

**Figure 1:**
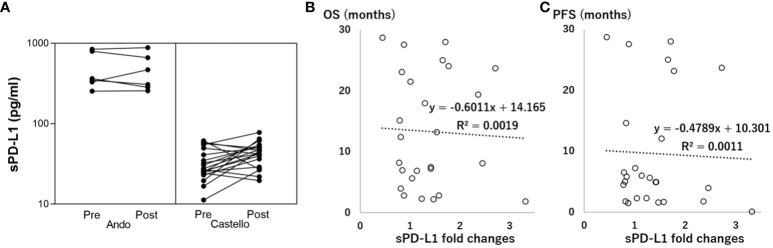
Association between soluble programmed death-ligand 1 (sPD-L1) fold changes and overall survival (OS/PFS, months). **(A)** Correlation between Pre-treatment (Pre) and Post-treatment (Post) sPD-L1. **(B)** Linear correlation between sPD-L1 fold changes and OS. **(C)** Linear correlation between sPD-L1 fold changes and PFS.

The pre/post-treatment serum changes in sPD-L1 concentration differed significantly between the two trials (**Table 1**, **Figure 1A**, 0.99 ± 0.21 vs. 1.51 ± 0.72, P=0.011). The overall pooled estimate indicated that sPD-L1 changes did not exhibit a linear correlation with OS (**Figure 1B**, r= -0.04, P=0.83)/PFS (**Figure 1C**, r= -0.03, P=0.87). This lack of correlation was consistent in both the trial by Ando et al. (OS; r= -0.44, P=0.38, PFS; r= -0.37, P=0.38) and the trial by Castello et al. (OS; r=0.036, P=0.88, PFS; r=0.0015, P=0.88).

**Figure 2:**
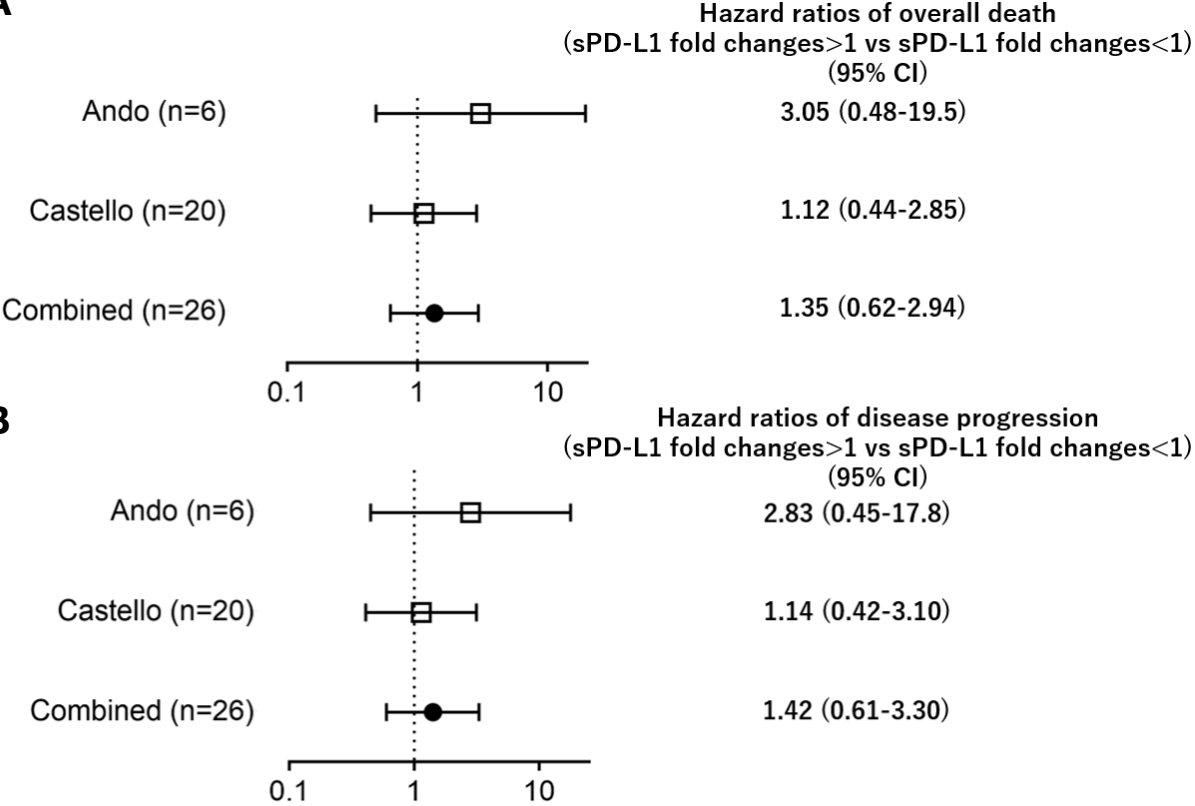
Forest plots representing hazard ratios of death **(A)** overall death, **(B)** disease progression) for sPD-L1 fold changes in each trial.

Additionally, increases in sPD-L1 (fold change>1) during PD-1 inhibitor treatment were not associated with OS/PFS when compared to decreases in sPD-L1 (fold change<1) (OS; HR=1.25; CI: 0.52–3.02, PFS; HR=1.42; CI: 0.61–3.30, **Figure 2**).

**Figure 3:**
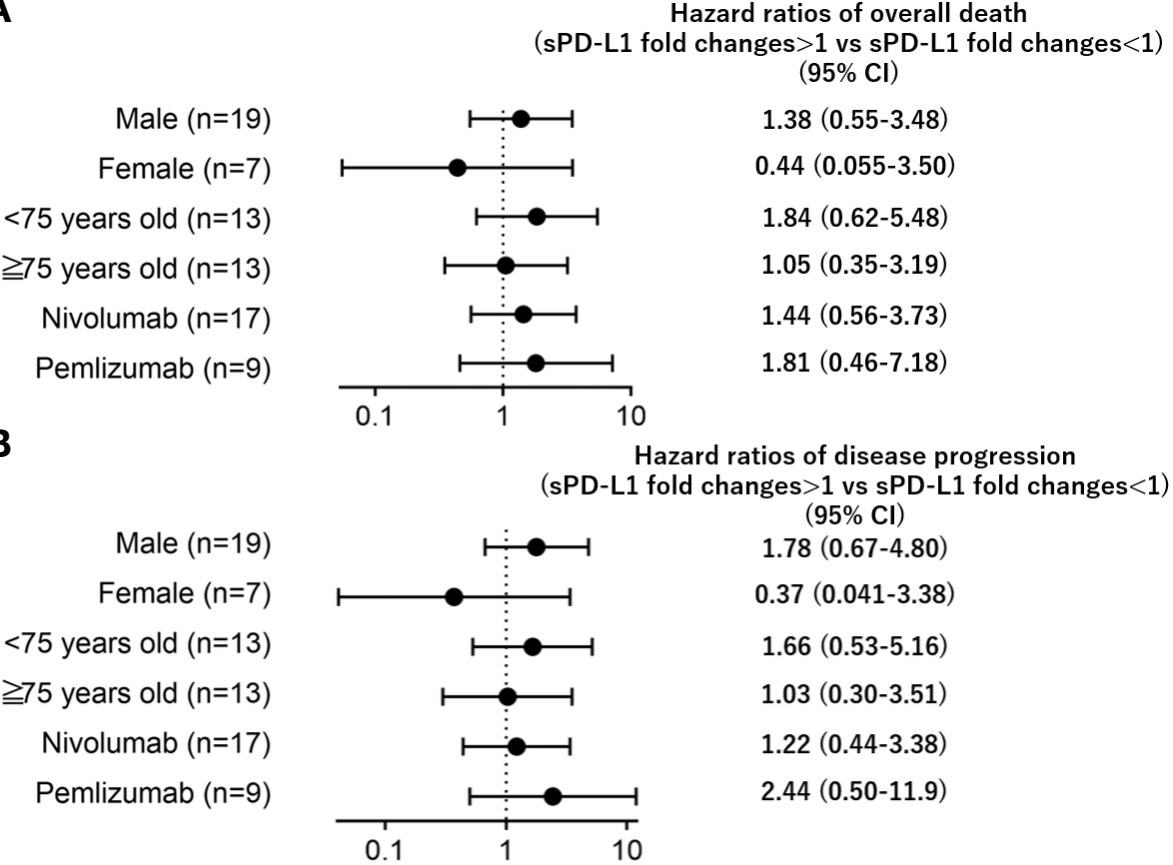
Forest plots representing hazard ratios of death **(A)** overall death, **(B)** disease progression) for sPD-L1 changes by subgroup.

Subgroup analyses of gender, age, or the type of treatment (nivolumab or pembrolizumab) revealed that the impact of sPD-L1 changes on overall mortality/disease progression remained consistent regardless (**Figure 3**).

## 4 Discussion

This individual patient data meta-analysis aimed to assess the prognostic significance of sPD-L1 changes during PD-1 inhibitor treatment in advanced NSCLC patients. Over the past few years, several studies have examined the association between pre-treatment or post-treatment sPD-L1 levels and prognosis in various cancers. In 2023, Széles et al. conducted a meta-analysis to evaluate the correlation between pre-treatment sPD-L1 and survival in a wide range of human malignancies. The pooled overall estimate indicated that sPD-L1 is a significant indicator of shorter OS/PFS in various cancers, with the strongest association observed in NSCLC. However, when it comes to sPD-L1 changes, previous studies have yielded inconsistent results, possibly due to small sample sizes or a lack of clinically meaningful relationships.

Recently, a large retrospective study by Himuro et al., involving 189 advanced NSCLC patients treated with immune checkpoint inhibitors (ICIs), including PD-1 inhibitors (nivolumab or pembrolizumab) or a PD-L1 inhibitor (atezolizumab), demonstrated a negative association between sPD-L1 changes during ICI therapy and OS. Notably, sPD-L1 changes were substantially higher with atezolizumab than with PD-1 inhibitors, potentially obscuring the specific effects of PD-1 inhibitors on prognosis (10).

In our meta-analysis, we examined the association between sPD-L1 changes and OS during PD-1 inhibitor treatment (**Figures 1**, **2**), and the results indicated no significant association. This finding contrasts with the previous study by Himuro et al., suggesting that sPD-L1 changes may not serve as predictive indicators for PD-1 inhibitor treatment, though they may do so for PD-L1 inhibitor treatment. Further investigation is needed to understand the underlying mechanisms behind sPD-L1 changes and their relationship with PD-1 inhibitor or PD-L1 inhibitor. This could involve laboratory-based studies to explore the biological and immunological factors that influence sPD-L1 levels.

While the study focused on sPD-L1 changes, there may be other potential biomarkers or combinations of biomarkers that could provide more accurate predictions of ICI efficacy. Orme et al. showed that a disintegrin and metalloprotease (ADAM)10 and ADAM17 cleave PD-L1 from the surface of malignant cells, thereby producing sPD-L1 that induces apoptosis in CD8+ T cells and compromises the killing of tumor cells by CD8+ T cells (15). These proteases also cleave soluble receptors, such as sCD30, sTNF-R1, sTNF-R2, and IL-2Ra, whose levels in plasma were correlated with sPD-L1 concentrations of advanced NSCLC patients (10, 16, 17). Future research should explore the utility of multiple biomarkers, including these soluble receptors, in combination.

sPD-L1 has been reported to be generated in and released from tumor cells and mature DCs (18). The biological activity of sPD-L1 remains unclear. In most cases, sPD-L1, like native PD-L1, binds to PD-1 to transmit negative regulatory signals for tumor immunity (19). Some studies have suggested that sPD-L1 may act as a PD-1 blocker, competitively suppressing the inhibitory effect of native PD-L1 (20, 21).

sPD-L1 is a type I transmembrane glycoprotein encoded by the CD274 gene. Transcription of this gene can produce multiple PD-L1 splice variants, which have varying lengths. Specifically, exon 4-enriched variants can generate a secreted form of PD-L1 in various cancers (19). According to the ELISA datasheet, the manufacturers only evaluate native PD-L1, not sPD-L1. Therefore, differences in the detection rate of sPD-L1 may be dependent on the ELISA method. To account for this variability, the analysis was conducted using the sPD-L1 fold change.

It remains unclear whether immune checkpoint inhibitor-related adverse events (irAEs), such as arthritis and myocarditis, are associated with sPD-L1 and fold changes. Himuro et al. has shown that sPD-L1 levels were highly correlated with soluble TNF receptors (sTNF-R1 and sTNF-R2) in plasma at baseline (10). Soluble TNF receptors can effectively prevent TNF from binding to its receptors, resulting in the prevention of immune adverse events. The bulk of the evidence also suggests that at least short courses of TNF inhibitors are safe in the treatment of irAEs. Thus, the levels of sPD-L1, sTNF-R1, and sTNF-R2 might be related to the prevention of irAEs.

One limitation of our analysis is that, despite our best efforts to obtain all available trial datasets, there are few reports on the continuous monitoring of sPD-L1, and acquiring individual patient data is challenging. Additionally, information on other factors such as patient characteristics, tumor size data, treatment duration, or other biomarkers, like tumor mutational burden or microsatellite instability, is not readily available and presents difficulties for analysis. It seems that serum sPD-L1 concentration may be correlated with these other factors. In future studies, investigating the correlations between pre- and post-treatment sPD-L1 levels and these additional biomarkers would offer further insights into the dynamics of PD-1 inhibitor treatments and patient outcomes.

This study has limitations related to the variability in cohort sizes among the included studies. Inconsistent results in the trial by Himuro et al. could also potentially impact our conclusions (10). The median follow-up period in the study was 10 months. To gain a more comprehensive understanding of the long-term effects of sPD-L1 changes, longer follow-up periods should be considered to assess the durability of the observed outcomes.

The strength of our study lies in being the first individual patient data meta-analysis focusing on the prognostic value of sPD-L1 changes during PD-1 inhibitor treatment. While this study included advanced NSCLC patients from Japan and Italy, expanding the research to more diverse patient populations, including those with different demographic and clinical characteristics, could provide a more comprehensive understanding of sPD-L1’s role.

## Conclusions

We found no evidence to support the prognostic significance of sPD-L1 changes during PD-1 inhibitor treatment in advanced NSCLC patients, regardless of sex, age, or the type of treatment administered. Consequently, we propose that sPD-L1 could serve as a valuable pre-treatment or post-treatment prognostic biomarker for immune checkpoint inhibitor (ICI) therapy when assessed at specific time points. However, continuous monitoring of sPD-L1 levels may not yield meaningful results.

Future research should explore whether specific subgroups of NSCLC patients may benefit from monitoring sPD-L1 levels. Identifying patient subpopulations that are more responsive to changes in sPD-L1 could lead to more personalized treatment strategies.

Investigating the potential role of sPD-L1 as a predictive biomarker for combination therapies involving PD-1 inhibitors and other treatments, such as chemotherapy or targeted therapies, could also be valuable.

## Data availability statement

Publicly available datasets were analyzed in this study. This data can be found here: doi:10.21873/anticanres.13716, doi:10.3390/cancers12061373.

## Author contributions

TS: Conceptualization, Data curation, Formal Analysis, Funding acquisition, Methodology, Project administration, Writing – original draft. EI: Formal Analysis, Writing – review & editing. RO: Validation, Writing – review & editing. SK: Supervision, Writing – review & editing. TT: Supervision, Writing – review & editing. SW: Conceptualization, Data curation, Formal Analysis, Funding acquisition, Supervision, Writing – review & editing.
