# Corrigendum: Soluble PD−L1 changes in advanced non-small cell lung cancer patients treated with PD-1 inhibitors: an individual patient data meta-analysis

**DOI:** 10.3389/fimmu.2023.1345513

**Published:** 2023-12-05

**Authors:** Takashi Shimizu, Eisuke Inoue, Ryotaro Ohkuma, Shinichi Kobayashi, Takuya Tsunoda, Satoshi Wada

**Affiliations:** ^1^ Department of Clinical Diagnostic Oncology, Clinical Research Institute for Clinical Pharmacology and Therapeutics, Showa University, Tokyo, Japan; ^2^ Clinical Research Institute for Clinical Pharmacology and Therapeutics, Showa University, Tokyo, Japan; ^3^ Showa University Research Administration Center, Showa University, Tokyo, Japan; ^4^ Division of Medical Oncology, Department of Medicine, School of Medicine, Showa University, Tokyo, Japan; ^5^ Department of Pharmacology, School of Medicine, Showa University, Tokyo, Japan; ^6^ Pharmacological Research Center, Showa University, Tokyo, Japan

**Keywords:** soluble PD-L1, advanced non-small cell lung cancer, PD-1 inhibitors, individual patient data meta-analysis, biomarker

In the published article, there was an error in the legend for **Figure 2** and **Figure 3** as published. **Figure 2B** and **Figure 3B**; Hazard ratios of **overall death**, and **Figure 3** legend; B; **disease** progression. The corrected legend appears below.

**Figure 2:**
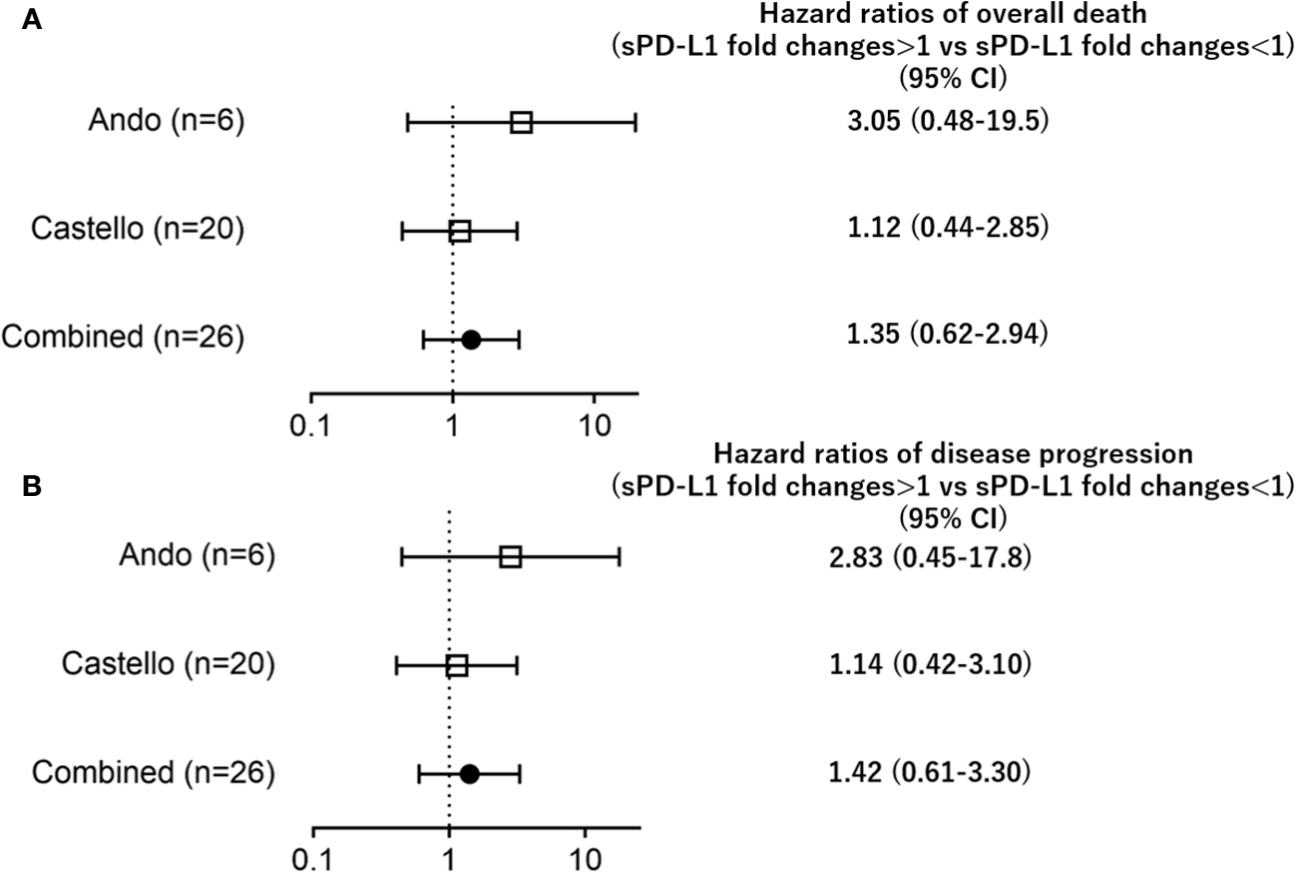
Forest plots representing hazard ratios of death (**A**; overall death, **B**; disease progression) for sPD-L1 fold changes in each trial.

**Figure 3:**
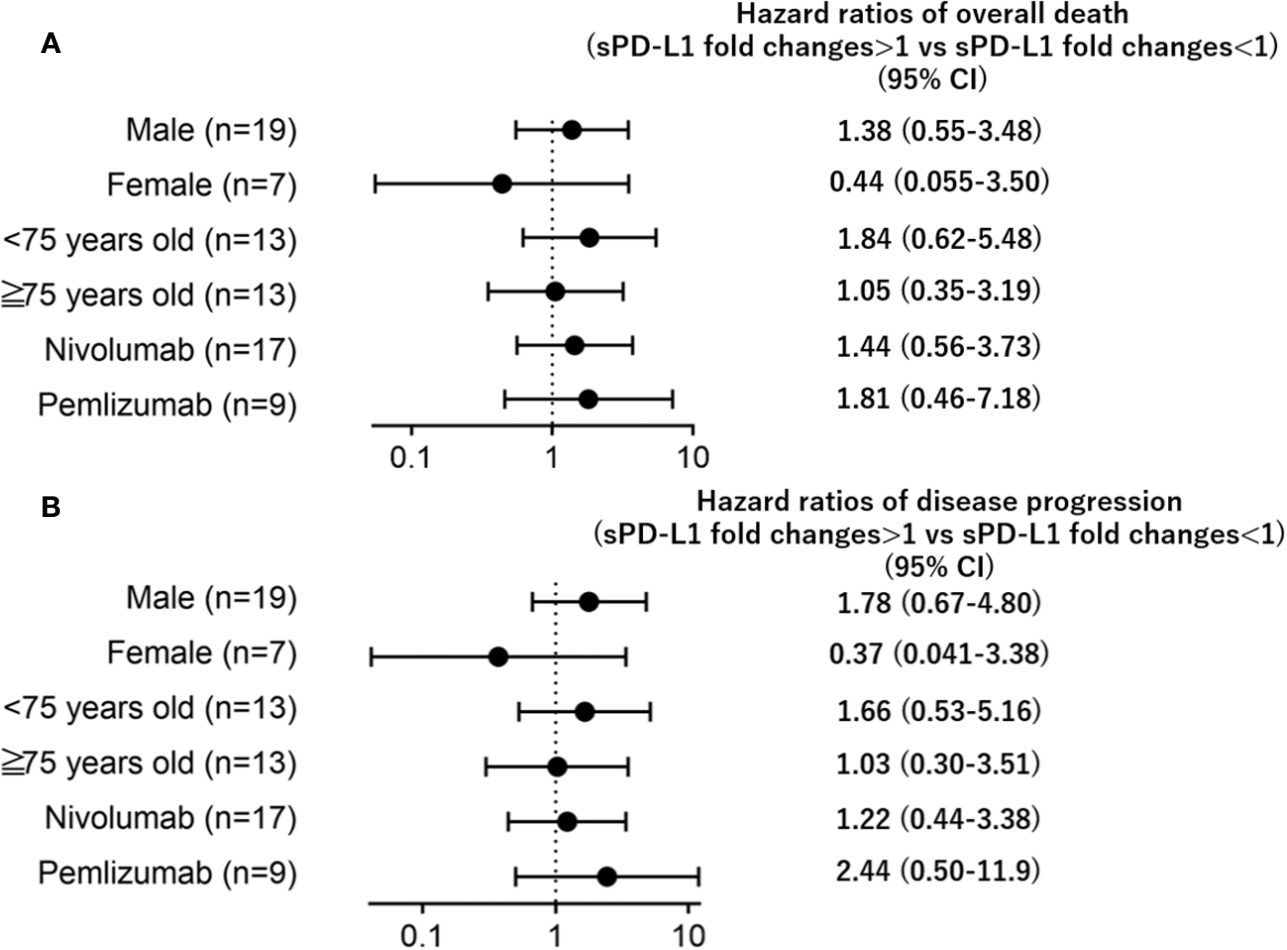
Forest plots representing hazard ratios of death (**A**; overall death, **B**; disease progression) for sPD-L1 changes by subgroup.

**Figure 2B** and **Figure 3B**; Hazard ratios of **disease progression**, and **Figure 3** legend; B; **disease** progression.

The authors apologize for these errors and state that this does not change the scientific conclusions of the article in any way. The original article has been updated.

